# Improving Quality and Compliance of Surgical Hand Scrubbing Practices: A Clinical Audit

**DOI:** 10.7759/cureus.80821

**Published:** 2025-03-19

**Authors:** Abubakr Muhammed, Mohmed Hussien Ahmed Mohmed, Ali Bashir Mohammed Ahmed, Mohamed Elfatih Osman Ali Abdelrahman, Romisaa Elamin, Musaab Ahmed Ali Fadul, Ibrahim Adil Hamadelniel Alhadi, Omer Abdellatif Ali Abdalla, Hind Abdelmoneim Mahjob Hajosman, Almoezldenalla Mahjub, Abdulaziz Alsamani Abdullah Omar, Mohey Aldien Ahmed Elamin Elnour, Mohamed Abdullah Jaffer Abdelrahman, Eilaf Eltayeb Abdalla Abdelgadir, Hussein Abu Obaida Abdelhadi Hussein, Seddig Alkazem Mohammed Alkheir, Mohamed Kamalaldein Hamad Mohamednour, Mozaffar Sirelkhatim Elzain Abdalla, Mustafa H Mohamed, Faris Jamalaldeen Mohammed Hamed

**Affiliations:** 1 Department of Surgery, University of Gezira, Madani, SDN; 2 Department of Medicine, University of Gezira, Madani, SDN; 3 Neurosurgery, Prince Othman Digna Teaching Hospital, Port Sudan, SDN; 4 Internal Medicine, Prince Othman Digna Teaching Hospital, Port Sudan, SDN; 5 General Surgery, Prince Othman Digna Teaching Hospital, Port Sudan, SDN; 6 Orthopaedic Surgery, Prince Othman Digna Teaching Hospital, Port Sudan, SDN; 7 Anaesthesia, Prince Othman Digna Teaching Hospital, Port Sudan, SDN; 8 Orthopaedic Surgery, Al-Neelain University, Khartoum, SDN

**Keywords:** audit cycle, compliance, hand hygiene, infection control, surgical site infections, who guidelines

## Abstract

Background

Surgical site infections (SSIs) are a critical concern in healthcare, particularly in developing countries, where they are among the most prevalent and challenging hospital-acquired infections. Adherence to proper hand hygiene practices is essential to prevent SSIs. However, compliance among surgical teams remains suboptimal due to factors such as time constraints, lack of training, and resource limitations. This study evaluates and enhances adherence to surgical hand scrubbing protocols at Osman Degna Teaching Hospital using World Health Organization (WHO) guidelines.

Methods

An observational cross-sectional audit was conducted in two cycles between August and October 2024, with 54 observations per cycle. Baseline adherence was assessed in the first cycle. Targeted interventions, including video demonstrations, hands-on training, and feedback, were implemented before the second cycle. Data were collected using a structured checklist and analyzed quantitatively to compare compliance rates and qualitatively to identify barriers to adherence.

Results

Compliance with hand scrubbing protocols improved significantly from 63.1% in the first cycle to 94.3% in the second. The most notable improvement (51.5%) was observed in rotational rubbing with clasped fingers. Other areas, including scrubbing palms and rinsing hands, showed substantial increases (30.3-42%). These findings highlight the effectiveness of structured training and feedback in enhancing adherence.

Conclusion

Targeted educational interventions significantly improved compliance with surgical hand scrubbing protocols, contributing to better infection control practices. While these improvements demonstrate the potential of training programs, continued efforts and long-term strategies are necessary to sustain progress and further reduce the risk of SSIs.

## Introduction

Surgical site infections (SSIs) are a significant concern in healthcare, particularly in developing countries, where they represent one of the most common and challenging hospital-acquired infections (HAIs) [1]. SSIs occur when bacteria or other pathogens infect the surgical site, leading to complications that can have serious consequences for patients. These infections are a major cause of increased patient morbidity and mortality, with affected individuals often requiring prolonged hospital stays, additional surgeries, or extended antibiotic treatments. In addition to the direct impact on patient health, SSIs contribute to significant financial burdens on healthcare systems [2]. The need for longer hospitalizations, extra medical procedures, and treatments places a strain on hospital resources and leads to increased healthcare costs for both the institution and the patient [2].

Despite the well-established importance of hand hygiene in preventing infections, studies show that only around 40% of healthcare professionals follow proper handwashing protocols [3]. Alcohol-based hand rubs have been introduced as an efficient and convenient alternative to traditional handwashing, offering faster sanitization and effective pathogen elimination. However, challenges persist in ensuring consistent compliance, due to factors like time constraints, lack of awareness, and insufficient resources. This non-compliance remains a major concern, especially in high-risk environments like surgery, where proper hand hygiene is crucial for preventing SSIs and other healthcare-associated infections. Addressing these barriers is essential to improving patient safety and infection control [3].

The use of alcohol-based hand sanitizers, which contain ethanol or isopropyl alcohol, has been shown to significantly reduce contamination by effectively eliminating microorganisms on healthcare workers' hands. These sanitizers offer a more efficient means of disinfection compared to non-medicated alternatives. However, monitoring and reporting healthcare-associated infections (HAIs) are critical for identifying trends and implementing appropriate infection control strategies. Unfortunately, the costs associated with comprehensive HAI surveillance can be prohibitively high, especially in resource-constrained settings, such as in many developing countries, making it a challenge for healthcare systems to maintain effective infection control measures [4].

General surgery residents and house officers in Sudan face difficulties in learning appropriate hand hygiene practices due to the operating room (OR) environment, including time limits, inadequate training, and a lack of skilled personnel. Even though following the World Health Organization's (WHO) hand hygiene recommendations is crucial for preventing infections, these obstacles make regular adherence challenging [5]. To strengthen these procedures and enhance patient safety, ongoing training, and assistance are crucial.

Given the persistent issue of low hand hygiene compliance in healthcare settings, particularly in surgical teams, this study seeks to evaluate the adherence of surgical personnel to hand hygiene protocols in a hospital setting. Through targeted interventions such as hands-on training, video demonstrations, and feedback, we aim to assess the effectiveness of these strategies in improving compliance and ultimately reducing the risk of SSIs. By exploring the factors influencing adherence and the effectiveness of educational interventions, this study hopes to contribute valuable insights into improving hand hygiene practices and patient safety in surgical settings.

## Materials and methods

Study design

This study was designed as an observational cross-sectional audit aimed at evaluating and enhancing adherence to surgical hand scrubbing and washing practices based on the World Health Organization (WHO) guidelines for hand scrubbing, ensuring compliance with international standards [5]. The audit was conducted in two cycles at Othman Digna Teaching Hospital between August 26, 2024, and October 20, 2024, with a sample size of 54 observations in each cycle.

Aim and objectives

The aim of this study is to evaluate and enhance infection control practices within the surgical team by assessing the consistency of adherence to hand scrubbing guidelines, identifying challenges faced by staff in maintaining proper hand scrubbing techniques and providing targeted recommendations or training to address identified gaps. Following the implementation of these measures, the study will reassess compliance and outcomes to determine the effectiveness of the interventions in supporting safer patient care.

Study area and population

The study was carried out in the operating theater of Othman Digna Teaching Hospital. The study population included all surgical operators and assistants who participated in surgical procedures during the audit period. This comprehensive inclusion ensured that the evaluation captured a broad representation of the team’s adherence to hand scrubbing protocols. A total of 54 observations were conducted in each audit cycle, providing a substantial sample to assess compliance levels across both phases of the study.

Data collection and analysis

Data was collected using a Google Form, which allowed for systematic and efficient documentation of observations and responses. The data collection was conducted in two phases: the first phase took place on 26th August 2024, and the second phase was conducted on 20th October 2024. The collected data were then subjected to both quantitative and qualitative analyses to gain a comprehensive understanding of hand scrubbing practices. For the quantitative analysis, compliance rates for each step of the hand scrubbing protocol were calculated, and overall adherence scores were compared between the two audit cycles to evaluate improvements. The qualitative analysis involved documenting challenges faced by staff, such as time constraints, lack of resources, or gaps in training. Patterns and trends in non-compliance were also identified to inform targeted interventions. Notably, the assessed participants were unaware that they were being observed, ensuring that their behaviors reflected genuine practices rather than being influenced by the presence of observers.

Comparison between audit cycles

During the first cycle, baseline adherence levels were recorded to identify areas for improvement. Between the cycles, staff were provided with targeted interventions, including video demonstrations, hands-on training sessions, and regular feedback on proper hand scrubbing techniques. The improvements observed in the second cycle were attributed to these focused educational interventions, which highlighted the positive impact of combining video demonstrations, practical training, and ongoing feedback in enhancing adherence to surgical hand hygiene protocols.

Ethical considerations

The audit was conducted in accordance with ethical guidelines for research involving human participants. Ethical approval for the Quality Improvement Project: ‘Improving Quality and Compliance of Surgical Hand Scrubbing Practices: A Clinical Audit’ was obtained from the Institutional Ethics Committee (IEC) of Othman Digna Teaching Hospital on 28th January 2024, with the approval number 2024/0163. This approval ensures that the project adheres to ethical guidelines and standards, protecting the rights and welfare of all participants involved. Participation in the audit was voluntary, and all data were collected anonymously to maintain the confidentiality and privacy of the surgical staff. Findings were used solely for quality improvement purposes.

## Results

In this study, 54 surgical participants were observed across two audit cycles to assess their adherence to hand scrubbing protocols. Initially, 63.1% of participants complied with the recommended hand hygiene guidelines during the first cycle. After receiving comprehensive training, which included video and live demonstrations, compliance significantly increased. By the second cycle, the success rate rose to 94.3%, showing a substantial improvement in adherence to the hand hygiene protocols.

The most notable improvement was observed in the criterion "rotational rubbing backwards and forwards with clasped fingers of the right hand into the left hand and vice versa," where compliance increased by 51.5%. Other areas also showed significant improvements: "rubbing fingertips on palms for both hands" saw a 37% increase, and "scrubbing palms with digits" improved by 38.6%. These areas are crucial for thorough hand hygiene and reducing the transmission of infections.

Additional improvements were seen in "scrubbing right palm over the back of the left hand and vice versa with fingers interlaced" (30.3% increase), and "rotational rubbing of the right thumb clasped in the left hand and vice versa" (38.6% increase). "Rinsing hands and arms by passing them through the water in one direction" also showed a 42% increase, demonstrating better adherence to this key step in preventing cross-contamination.

Furthermore, compliance in "holding the hands above the elbow at all times" improved by 20%, and the avoidance of "splashing water onto the dress" showed an 18.6% increase. The "do not move the arms back and forth through the water" criterion saw a 26.3% improvement, indicating increased awareness of proper techniques for maintaining aseptic conditions.

Overall, the training interventions resulted in significant improvements in all aspects of hand hygiene, contributing to a higher level of compliance and more effective infection control in the surgical setting. This highlights the importance of continuous training and practical demonstrations to reinforce best practices for infection prevention. Table 1 shows the results in the first and second cycles and the percentage of improvement.

**Table 1:** A comparison between first and second cycle in terms of adherence to hand scrubbing protocols

Items of Scrub	First Cycle N (%)	Second Cycle N (%)	Improvement (%)
Wet the hands and forearms	49 (90.0%)	54 (100.0%)	10.00%
Rub palm to palm finger interlaced	29 (54.0%)	50 (92.6%)	38.60%
Scrub right palm over back of left hand and vice versa with fingers interlaced	36 (66.0%)	52 (96.3%)	30.30%
Rotational rubbing backwards and forwards with clasped finger of right hand into left hand and vice versa	16 (30.0%)	44 (81.5%)	51.50%
Rotational rubbing of right thumb clasped in left hand and vice versa	29 (54.0%)	50 (92.6%)	38.60%
Rub fingertips on palms for both hands	27 (50.0%)	47 (87.0%)	37.00%
Continue with rotational action down opposing arms working to the elbow for 1 minute	37 (68.0%)	53 (98.1%)	30.10%
Rinse hands and arms by passing them through the water in one direction, only from fingertips to elbow	31 (58.0%)	54 (100.0%)	42.00%
Do not move the arms back and forth through the water	38 (70.0%)	52 (96.3%)	26.30%
Do not splash the water onto the dress	40 (74.0%)	50 (92.6%)	18.60%
Hold the hands above the elbow at all times	43 (80.0%)	54 (100.0%)	20.00%
Mean compliance	34 (63.1%)	51 (94.3%)	31.20%

## Discussion

This study demonstrated a significant improvement in hand hygiene compliance among surgical team members following targeted educational interventions. The baseline compliance rate of 63.1% in the first audit cycle improved to 94.3% in the second cycle after the implementation of video demonstrations, hands-on training, and regular feedback. The most notable improvement was observed in the criterion of rotational rubbing with clasped fingers, which saw a 51.5% increase in compliance. Other critical steps, such as scrubbing palms and rinsing hands, also showed substantial improvements, ranging from 30.3% to 42%. These findings highlight the effectiveness of structured training and feedback in enhancing adherence to surgical hand hygiene protocols. Handwashing remains a cornerstone of infection control in healthcare settings, with its importance underscored by numerous studies demonstrating its effectiveness in reducing the spread of nosocomial infections and healthcare-associated infections (HAIs) [6-8]. The importance of hand hygiene in healthcare settings has been well-documented in the literature.

Al Sawafi KM (2021) emphasized the role of hand hygiene policies and patient safety culture in improving healthcare workers' adherence to hand hygiene practices, particularly in critical care settings [9]. Their scoping review highlighted the need for continuous awareness programs, adequate resources, and comprehensive monitoring to sustain high levels of compliance. Our findings align with their recommendations, as the targeted interventions in our study, including hands-on training and feedback, significantly improved compliance rates. This further underscores the importance of structured educational programs in fostering a culture of safety and adherence to hand hygiene protocols [9].

One of the main ways that infections spread is through healthcare personnel's contaminated hands. Therefore, practicing good hand hygiene is essential for preventing the spread of infections, which in turn lowers the risk of infection, medical expenses, hospital stays, and reimbursement rates. According to the Centers for Disease Control and Prevention (CDC) [10], hand cleanliness is still the best way to stop illnesses from spreading in medical settings.

Surgical hand antisepsis requires a specific set of skills that go beyond routine handwashing to ensure thorough decontamination before surgery [11]. Surgical site infections (SSIs) are one of the most common types of hospital-acquired infections, often resulting from the unintentional transfer of harmful microorganisms to a patient's surgical site [12].

Our study, along with others, highlights the significant impact of targeted interventions, such as hands-on training and feedback, in improving compliance with surgical hand hygiene protocols. The baseline compliance rate of 63.1% in our study improved to 94.3% after interventions, with notable improvements in critical steps such as rotational rubbing and scrubbing fingertips. These findings are consistent with those of Yousif Mohamed et al., who reported a similar increase in compliance from 64.14% to 93.94% following similar interventions [13]. Additionally, Mukherjee et al. (2021) demonstrated that repeated training sessions could achieve 100% compliance among surgical internees, further emphasizing the importance of continuous education and reinforcement of hand hygiene practices [14]. Collectively, these studies reinforce the necessity of structured, evidence-based interventions to enhance hand hygiene compliance, ultimately reducing the risk of SSIs and improving patient outcomes. As Gök et al. (2016) noted, the choice of antiseptic solutions and techniques in surgical handwashing remains a topic of debate, but the consistent improvement in compliance across studies suggests that the method of intervention may be as critical as the choice of antiseptic [15]. Future research should continue to explore the most effective strategies for sustaining high levels of hand hygiene compliance in diverse healthcare settings.

The evidence from these studies reinforces the idea that ongoing education and training are essential for maintaining high levels of hand hygiene compliance. Moreover, the improvements observed in both our study and others highlight the effectiveness of multi-modal approaches, which combine various educational methods to ensure that healthcare workers consistently follow hand hygiene protocols. Such improvements are crucial for reducing the incidence of healthcare-associated infections, particularly SSIs, and for enhancing patient safety overall.

Limitations and recommendations

While this study demonstrated promising results in improving hand hygiene compliance, several limitations must be considered. The research was conducted at a single institution with a small sample size of 54 participants per audit cycle, limiting the generalizability and statistical power of the findings. Additionally, the absence of a control group makes it difficult to determine the exact impact of the intervention. The short duration of the study also prevents evaluation of the long-term sustainability of improvements. Factors such as prior training, personal habits, workload, and institutional support were not controlled, which could have influenced the results.

To address these limitations, future studies should involve multiple centers with larger sample sizes and employ randomized controlled trials to strengthen the evidence. Longer follow-up periods are necessary to assess the sustainability of improvements and their impact on SSIs. Re-audits, varied educational methods, and integration of hand hygiene training into professional development programs can further enhance adherence. Healthcare institutions should allocate resources for proper hand hygiene supplies and foster a culture of safety and accountability. Finally, exploring the effects of external factors like workload and staffing on hand hygiene compliance could help tailor more effective interventions.

## Conclusions

In conclusion, our study demonstrated a significant improvement in hand hygiene compliance among surgical team members following targeted educational interventions. The results highlight the effectiveness of video demonstrations, hands-on training, and regular feedback in improving adherence to surgical hand hygiene protocols. While there are limitations to the study, the findings suggest that such interventions can be an effective strategy for reducing the risk of surgical site infections and improving patient outcomes. Further research is needed to explore the long-term sustainability of these improvements and to identify additional factors that may impact hand hygiene compliance.
